# Tissue Microbiome Profiling Identifies an Enrichment of Specific Enteric Bacteria in *Opisthorchis viverrini* Associated Cholangiocarcinoma

**DOI:** 10.1016/j.ebiom.2016.04.034

**Published:** 2016-05-06

**Authors:** Kern Rei Chng, Sock Hoai Chan, Amanda Hui Qi Ng, Chenhao Li, Apinya Jusakul, Denis Bertrand, Andreas Wilm, Su Pin Choo, Damien Meng Yew Tan, Kiat Hon Lim, Roy Soetinko, Choon Kiat Ong, Dan G. Duda, Simona Dima, Irinel Popescu, Chaisiri Wongkham, Zhu Feng, Khay Guan Yeoh, Bin Tean Teh, Puangrat Yongvanit, Sopit Wongkham, Vajaraphongsa Bhudhisawasdi, Narong Khuntikeo, Patrick Tan, Chawalit Pairojkul, Joanne Ngeow, Niranjan Nagarajan

**Affiliations:** a Genome Institute of Singapore, 138672, Singapore; b Division of Medical Oncology, National Cancer Centre Singapore, 11 Hospital Drive, 169610, Singapore; c Laboratory of Cancer Epigenome, National Cancer Centre Singapore, 11 Hospital Drive, 169610, Singapore; d Department of Gastroenterology and Hepatology, Singapore General Hospital, Outram Road, 169608, Singapore; e Department of Pathology, Singapore General Hospital, Outram Road, 169608, Singapore; f Cancer Science and Stem Cell Biology, Duke-NUS Graduate Medical School, 8 College Road, 169857, Singapore; g Edwin L. Steele Laboratories for Tumor Biology, Department of Radiation Oncology, Massachusetts General Hospital and Harvard Medical School, Boston, MA, USA; h Center of Digestive Diseases and Liver Transplantation, Fundeni Clinical Institute, Bucharest, Romania; i Liver Fluke and Cholangiocarcinoma Research Center, Faculty of Medicine, Khon Kaen University, Khon Kaen 40002, Thailand; j Dept. of Medicine, Yong Loo Lin School of Medicine, National University of Singapore, Singapore; k Dept. of Gastroenterology and Hepatology, National University Health System, Singapore; l Oncology Academic Clinical Program, Duke-NUS Graduate Medical School, 8 College Road, 169857, Singapore

**Keywords:** Microbiome, Cancer, Cholangiocarcinoma, Liver fluke

## Abstract

Cholangiocarcinoma (CCA) is the primary cancer of the bile duct system. The role of bile duct tissue microbiomes in CCA tumorigenesis is unestablished. To address this, sixty primary CCA tumors and matched normals, from both liver fluke (*Opisthorchis viverrini*) associated (OVa, n = 28) and non-*O. viverrini* associated (non-OVa, n = 32) cancers, were profiled using high-throughput 16S rRNA sequencing. A distinct, tissue-specific microbiome dominated by the bacterial families *Dietziaceae*, *Pseudomonadaceae* and *Oxalobacteraceae* was observed in bile duct tissues. Systemic perturbation of the microbiome was noted in tumor and paired normal samples (vs non-cancer normals) for several bacterial families with a significant increase in *Stenotrophomonas* species distinguishing tumors vs paired normals. Comparison of parasite associated (OVa) vs non-associated (non-OVa) groups identified enrichment for specific enteric bacteria (*Bifidobacteriaceae*, *Enterobacteriaceae* and *Enterococcaceae*). One of the enriched families, *Bifidobacteriaceae*, was found to be dominant in the *O. viverrini* microbiome, providing a mechanistic link to the parasite. Functional analysis and comparison of CCA microbiomes revealed higher potential for producing bile acids and ammonia in OVa tissues, linking the altered microbiota to carcinogenesis. These results define how the unique microbial communities resident in the bile duct, parasitic infections and the tissue microenvironment can influence each other, and contribute to cancer.

## Highlights

• *Stenotrophomonas*, implicated in bile duct infections, is enriched in tumor tissues of non-fluke related cholangiocarcinoma.
• *O. viverrini* infection may alter composition of bile duct tissue microbiomes.
• Enteric bacteria with metabolic outputs linked to carcinogenesis are enriched in *O. viverrini* associated tissue microbiomes.

The link between microbiota and cancer of the gastrointestinal (GI) tract has been extensively studied. However, the role of tissue microbiome in cholangiocarcinoma (CCA), cancer of the bile duct (an organ connected to the GI tract), is largely unknown. In this study, we detected intriguing compositional differences in the tissue microbiomes of liver fluke related and non-related CCA. Taken together, our data suggests a connection between parasitic infections, tissue microbiome alterations, tissue micro-environment changes and CCA development.

## 1 Introduction

Cholangiocarcinoma (CCA) is a tumor that manifests from the malignant transformation and uncontrolled proliferation of biliary tree epithelial cells known as cholangiocytes (Lazaridis and Gores, 2005). Currently, CCA patients have poor prognosis and therapeutic options have limited efficacy. CCA incidence fluctuates according to varying geographical regions where underlying risk factors differ (Tyson and El-Serag, 2011). Strikingly, there can be a large variation in incidence rates even among different regions in the same country. In northeast Thailand where 85% of total primitive liver cancers are in the form of CCA, incidence rates can reach as high as 85 in 100,000 (Bragazzi et al., 2012). In comparison, the north, central and south of Thailand have much lower CCA incidence rates at 14.6/100,000, 14.4/100,000 and 5.7/100,000 respectively, correlating with the prevalence of liver fluke (*Opisthorchis viverrini*) infection in the region (Bragazzi et al., 2012). On the other hand, choledochal cysts, hepatolithiasis and primary sclerosing cholangitis are the main risk factors for CCA in locations where liver fluke parasitism is not prevalent (Tyson and El-Serag, 2011). Although the clinical risk factors for CCA have been clearly delineated, the molecular mechanisms underlying the different risk factors for CCA development still remain unclear (Fava, 2010).

The interaction of microbes with host cells is known to have a major impact on the health of the host (Hooper et al., 2012, Kau et al., 2011) and has been implicated in diseases ranging from metabolic disorder (Qin et al., 2012) to cancer (Schwabe and Jobin, 2013, Sobhani et al., 2011, Nagarajan et al., 2012). Specifically, there is an increasing interest in understanding the role of the tissue microbiome in carcinogenesis, with extensive work primarily in the area of colon cancer (Kostic et al., 2013, Kostic et al., 2012). The bile duct is an important component of the digestive system carrying bile fluids from the liver and gall bladder to the intestine. Despite the bile duct's proximity to the large microbial reservoir in the gastrointestinal tract, and its susceptibility to microbial infection (Carpenter, 1998), surprisingly little is known about the human bile duct microbiome (Brook, 1989, Olsson et al., 1998) and its association with CCA. This could partly be attributed to technical difficulties in the acquisition of a large enough cohort (CCA is a relatively rare cancer) and in profiling microbial abundances from small biopsies with low bacterial load (Lluch et al., 2015).

To comprehensively characterize the bile duct tissue microbiome, 131 samples were profiled using 16S rRNA sequencing, including 60 tumor-normal pairs from CCA patients and 11 control samples (bile fluids, gastric and hepatic tissue). Bile duct tissues were observed to harbor a distinct microbiome, compared to bile fluids, gastric and hepatic tissues, dominated by the bacterial families *Dietziaceae*, *Pseudomonadaceae* and *Oxalobacteraceae*. Unlike in colorectal cancer, CCA cases exhibited a shared systemic perturbation in both tumor and adjacent normal tissues, with significant focal tumor vs normal differences restricted to opportunistic pathogens from the genus *Stenotrophomonas*. Distinct mutational patterns in liver fluke associated and non-associated CCA were previously observed (Chan-On et al., 2013, Ong et al., 2012), linked to their underlying etiologies and risk factors. To understand the contribution of the tissue microbiome, CCA patients from both liver fluke (*O. viverrini*) associated (OVa group, n = 28) and non-*O. viverrini* associated (non-OVa group, n = 32) groups were profiled. Comparison across groups revealed the enrichment of enteric bacteria belonging to the families *Bifidobacteriaceae*, *Enterobacteriaceae* and *Enterococcaceae*, some of which could have been directly introduced into the bile duct by the parasite. Inference of functional capacities of CCA microbiomes revealed a higher potential for producing bile acids and ammonia in OVa tissues, linking the altered microbiota to carcinogenesis (Debruyne et al., 2001, Tsujii et al., 1992).

## 2 Materials and Methods

### 2.1 Clinical Specimens

Sixty primary tumor and adjacent matched normal samples (non-neoplastic liver) were obtained from the Singhealth Tissue Repository (Singapore), Srinagarind Hospital Khon Kaen University (Thailand) and the Fundeni Clinical Institute (Romania). Selected samples were subjected to pathology review to confirm that matched normal samples did not contain tumor cells. All samples were collected with signed informed consent from patients and study approved by SingHealth Centralized Institutional Review Board (2006/449/B and 2008/456/B), NHG Domain Specific Review Board (2000/00329), Ethics Committee of the Clinical Institute of Digestive Diseases and Liver Transplantation Fundeni (215/18.01.2010) and Human Research Ethics Committee at Khon Kaen University (HE471214). Five non-cancer hepatic tissues and two bile fluid samples were obtained from the NUHS Tissue repository. Four non-cancer gastric mucosa samples were collected under a study approved by NHG Domain Specific Review Board (Ref: 2000/00329). Clinicopathological information for subjects including age, gender, ancestry and tumor subtype was reviewed retrospectively (Supplementary File 1).

### 2.2 DNA Extraction

Tissue samples were thawed on ice and were transferred into Lysing Matrix E tubes (MP Biomedicals, Solon, U.S.A.). A volume of 650 μl of ATL Buffer (Qiagen, Hilden, Germany) was added to each sample. Biliary fluid samples were first centrifuged at 8000*g* for 15 min at 4 °C. The supernatant was discarded and 650 μl of ATL Buffer was added to re-suspend the cell pellet before transferring into Lysing Matrix E tubes. Both tissue and bile fluid samples were then subjected to bead-beating with FastPrep-24 Instrument (MP Biomedicals, Solon, U.S.A.) at a speed of 6.0 m/s for 70 s. Following that, the samples were centrifuged at 16,000*g* for 5 min and 30 μl of Proteinase K (Qiagen, Hilden, Germany) was added to the supernatant. Samples were then incubated at 56 °C for 15 min. Isolation of DNA was carried out using the EZ1 DNA Tissue Kit (Qiagen, Hilden, Germany) along with the automated EZ1 Advanced XL Instrument (Qiagen, Hilden, Germany). Purified DNA was quantified with Qubit dsDNA HS Assay Kit (Life Technologies, Eugene, U.S.A.) and stored at − 20 °C.

### 2.3 16S rRNA Gene Amplification

16S rRNA polymerase chain reaction (PCR) amplification was performed as previously described (Ong et al., 2013). Briefly, two hundred nanograms of extracted DNA was amplified using primers that target the V3 to V6 region of the 16S rRNA gene. The primer sequences that were used for 16S rRNA PCR amplification are 338_F: ACT CCT ACG GGA GGC WGC and 1061_R: CRR CAC GAG CTG ACG AC. HotStar HiFidelity Polymerase Kit (Qiagen, Hilden, Germany) was used for PCR and was performed according to the manufacturer's manual except for a modification in primer concentrations (0.5 μM) and the addition of MgSO_4_ at a final concentration of 2 mM. PCR was set up with the following conditions: Initial denaturation at 95 °C for 5 min, followed by 35 cycles of denaturation at 95 °C for 30 s, annealing at 59 °C for 30 s and extension at 72 °C for 1 min. Lastly, PCR was completed with a step of final extension at 72 °C for 6 min. Agencourt AMPure XP (Beckman Coulter, Brea, U.S.A.) was used to purify the amplified products and purified products were visualized using Agilent Bioanalyzer, prepared with Agilent High Sensitivity DNA Kit (Agilent Technologies, Waldbronn, Germany). As controls for assay specificity, 16S rRNA PCR was performed with extraction controls and the absence of amplification products was confirmed using Agilent Bioanalyzer.

### 2.4 Library Construction

A standardized amount of 500 ng of PCR product was subjected to shearing using Adaptive Focused Acoustics™ (Covaris, Woburn, U.S.A.). Fragment sizes ranged from 100 to 400 bp. DNA libraries were built using Gene Read DNA Library I Core Kit (Qiagen, Hilden, Germany) and were processed according to the manufacturer's protocol except for using barcode adaptors in place of the recommended adapter set. DNA libraries were enriched using custom index-primers that would tag each sample with an index. The enrichment protocol was adapted from Multiplexing Sample Preparation Oligonucleotide kit (Illumina, San Diego, U.S.A.). Quantification of libraries was carried out using Agilent Bioanalyzer, prepared with Agilent High Sensitivity DNA Kit (Agilent Technologies, Waldbronn, Germany). An Illumina HiSeq2000 instrument was used to perform paired-end sequencing (2 × 101 bp or 2 × 75 bp reads) on all DNA libraries built.

### 2.5 Preprocessing of Sequencing Reads and 16S rRNA Profiling

Sequenced bases were trimmed off at the 3′ ends of reads, starting at bases with quality scores < 3. Only read pairs with both reads longer than 60 bp were kept. 16S reads were identified by mapping them to the 16S rRNA database (Pruesse et al., 2007) provided in EMIRGE (Miller et al., 2011) using BWA-MEM (Li and Durbin, 2009) and with default parameters (0.7.9a). A mapping was considered valid only if at least 80% of the bases matched in at least one of the reads in a pair. Read mappings were used to determine relative abundance of taxa as previously described (Ong et al., 2013). Briefly, EMIRGE (Miller et al., 2011), a probabilistic expectation-maximization based algorithm, was used to reconstruct and measure the abundances of the 16S rRNA sequences. The reconstructed sequences were then taxonomically classified by using BLAST to compare them to the NR database. The distribution of the number of 16S sequencing reads available for the samples in the cohort is detailed in Supplementary Fig. 1.

### 2.6 Computing Diversity Metrics

Shannon diversity index (H) is a metric commonly used in ecology for measuring the diversity of a community. For each sample we calculated the Shannon diversity index given as $H=-\sum_{i=1}^{N}p_{i}\ln p_{i}$ using a custom R script where N is the number of families in each sample and p_i_ is the relative proportion of a specific family i. Yue-Clayton theta (*θ*) index (Yue and Clayton, 2005) was used for quantifying similarity across microbiomes. The index was also calculated with a custom R script using the formula, $\theta =\frac{\sum_{i}p_{i\;}q_{i}}{\sum_{i}{\left(p_{i}-q_{i}\right)}^{2}+\sum_{i}p_{i}q_{i}}$, where p_i_ and q_i_ are relative proportions of a specific family i, in sample p and sample q respectively. Distances between microbial communities was computed using weighted and unweighted UniFrac distances (Lozupone and Knight, 2005) and tested for statistical significance with the Adonis test script in QIIME (Caporaso et al., 2010).

### 2.7 Assessing Differential Abundance of Bacterial Taxa

The non-parameteric Wilcoxon rank sum test (for unpaired samples) and the Wilcoxon signed rank test (for paired samples) were used to test for differences in bacterial abundance. Correction for multiple hypothesis testing was done by computing false discovery rates using the R function “p.adjust”. Bacterial taxa with mean abundance lower than 0.5% (across all samples) were excluded from differential abundance analysis.

### 2.8 Whole Metagenome Profiling of the Liver Fluke Microbiome

Liver fluke genomic reads were obtained from a previous study (Young et al., 2014) (pool of  > 10 flukes). Sequencing reads were preprocessed using the NGS QC Toolkit (Patel and Jain, 2012) (version 2.2.3, default parameters) to filter contamination from Illumina read adaptors (keeping only pairs where both reads were unfiltered). The liver fluke microbiome was then profiled using the program MetaPhlAn (Segata et al., 2012) (default parameters) with the filtered reads.

### 2.9 Microbiome Functional Analysis

We used the program PICRUSt (Langille et al., 2013) (version 1.0.0) to predict functional metagenomic content from the 16S marker gene data. Specifically, the EMIRGE (Miller et al., 2011) reconstructed amplicon sequences and abundances were converted to QIIME (Caporaso et al., 2010) format and each amplicon sequence was duplicated according to relative abundances reported by EMIRGE. We picked OTUs at 90% similarity (between the genus and family identity threshold (Yarza et al., 2014)) against Greengenes (McDonald et al., 2012) reference OTUs (gg_13_5_otus.tar.gz, provided along with the PICRUSt package) with QIIME's pick_closed_reference_otus.py script (version 1.8), following the closed reference OTU picking protocol. The output OTU table was then normalized by 16S copy numbers with PICRUSt's normalize_by_copy_number.py script. The final metagenome prediction was produced using PICRUSt's predict_metagenomes.py script. The predicted gene abundances were analyzed using HUMAnN (Abubucker et al., 2012) (version 0.99) to estimate pathway abundances. Differential abundance testing was performed using the Wilcoxon rank-sum test.

### 2.10 Data Deposition

All 16S rRNA sequencing reads are available from the NCBI short read archive (SRA) under the bioproject number PRJNA297250. Liver fluke genomic reads (Young et al., 2014) are available from run ID SRR2529483.

## 3 Results

### 3.1 Diversity and Distinctness of Human Bile Duct Tissue Microbiome in CCA Patients

**Fig. 1:**
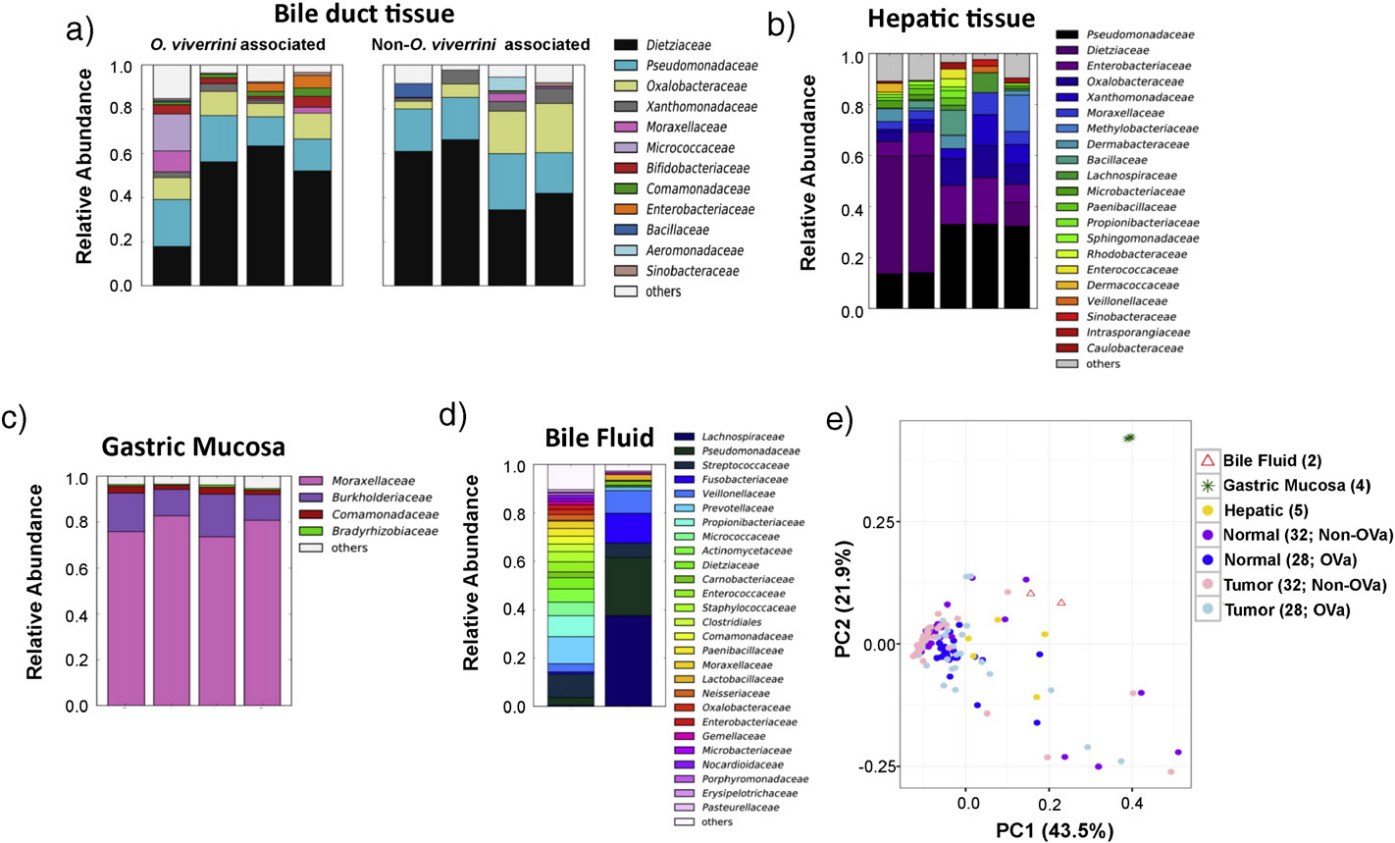
Tissue microbiome 16S rRNA profiles of the bile duct, gastric mucosa and liver. Barplots showing microbiome profiles of (a) bile duct tissue (4 out of 28 OVa and 4 out of 32 non-OVa) from CCA patients, (b) non-cancer hepatic tissue (n = 5), (c) non-cancer gastric mucosa (n = 4) and (d) bile fluid (n = 2) from CCA patients at family level resolution. Only families with mean relative abundance > 0.5% are shown. (e) Principle coordinates analysis based on Jensen-Shannon distance of the microbiome profiles (family level) of different tissue types (OVa: *O. viverrini* associated tissue).

The bacterial families, *Dietziaceae*, *Pseudomonadaceae* and *Oxalobacteraceae* were found to be the major inhabitants of bile duct tissues in CCA patients (Fig. 1a; showing 4 representative OVa and non-OVa profiles). *Pseudomonadaceae* and *Oxalobacteraceae* have previously been reported to be dominant in the penis foreskin microbiome (Price et al., 2010). *Dietzia* are metabolically versatile and were previously found in the human skin and oral microbiome (Dewhirst et al., 2010, Koerner et al., 2009). Comparing the hepatic tissue microbiome of non-CCA subjects (n = 5) with bile duct tissue microbiomes (from CCA patients; n = 60) revealed several shared families (e.g. *Dietziaceae*, *Pseudomonadaceae* and *Oxalobacteraceae*; Fig. 1b). This is consistent with the fact that part of the biliary tree is embedded within the liver. In contrast, gastric mucosal tissue microbiomes (n = 4) were found to be clearly distinct from bile duct tissue microbiomes (Adonis test *p*-value = 0.001 for unweighted and weighted UniFrac distances) and dominated by *Moraxellaceae*, with lower abundances of *Burkholderiaceae* (Fig. 1c). These findings are consistent with current culture-based understanding of these tissues: *Pseudomonas* species have been frequently isolated from bile samples (Elomari et al., 1997, Sutter, 1968) while *Acinetobacter* species (*Moraxellaceae* family) have been reported to colonize gastric tissue and cause gastritis (Rathinavelu et al., 2003). Analysis of bile fluid samples (n = 2) showed that while *Pseudomonadaceae* was the main microbial component, there was substantial variability between the two samples, high diversity in one sample and a composition that deviates notably from bile duct (n = 60; Adonis test *p*-value = 0.001 and 0.009 for unweighted and weighted UniFrac distances, respectively) and liver tissue microbiomes (n = 5; Adonis test *p*-value = 0.044 and 0.001 for unweighted and weighted UniFrac distances, respectively; Fig. 1d and e). These observations indicate that bile fluids may not be a reliable proxy for studying the resident microbiome of bile duct tissues, though further studies with matched samples are needed to confirm this. Overall, bile duct tumor tissue microbiomes from CCA patients exhibited substantial variability but clustered with adjacent normals, and were distinct from gastric tissues microbiomes (Fig. 1e; Supplementary Fig. 2).

### 3.2 Enrichment of Specific Bacterial Taxa and Their Association With CCA Etiology

**Fig. 2:**
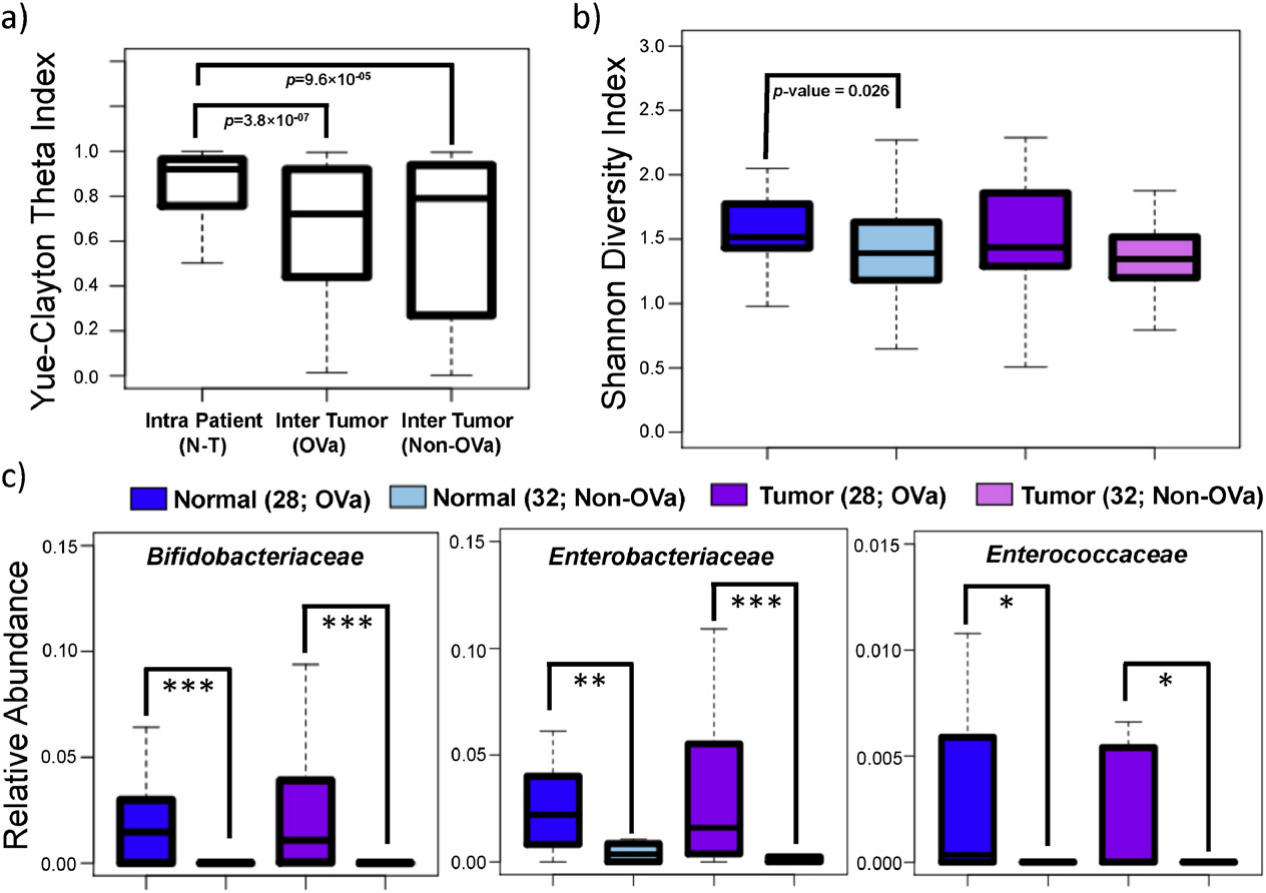
Comparison of *O. viverrini* associated and non-associated paired tumor-normal tissue microbiomes. (a) Boxplots of Yue-Clayton theta indices for quantifying similarity across tissue microbiomes (family level) within tumor-normal pairs, for *O. viverrini* associated (n = 28; OVa) tumors and non-*O. viverrini* associated (n = 32; non-OVa) tumors respectively. (b) Boxplots depicting the microbiome diversity of *O. viverrini* associated and non-associated tumor and adjacent normal tissues. (c) Boxplots showing the relative abundance of the 3 families identified to be enriched in *O. viverrini* associated (n = 28) vs non-*O. viverrini* (n = 32) associated tissues. All *p*-values were calculated using the Wilcoxon rank-sum test, where ***, ** and * represent FDR adjusted *p*-values < 0.01, 0.05 and 0.1 respectively.

Microbiome compositional differences in CCA were investigated based on 16S rRNA profiling on paired tumor-normal tissues for 32 non-*O. viverrini* associated (non-OVa group) and 28 *O. viverrini* associated (OVa group) CCA patients (Supplementary File 1 and Supplementary Figs. 3 and 4). The non-OVa and OVa groups were approximately matched for age (mean = 56.6 vs 57.9), gender (15 out of 32 male vs 19 out of 28 male) and anatomical subtype (8 out of 32 extra-hepatic vs 8 out of 28 extra-hepatic) but not for ethnicity (non-thai vs thai; Supplementary File 1). Intra patient (tumor vs normal) microbiome profiles were found to be more similar relative to inter-tumor (across patient) microbiome profiles (OVa group *p*-value = 3.8 × 10^− 07^; non-OVa group *p*-value = 9.6 × 10^− 05^; Fig. 2a). This suggests the existence of an individual-specific tissue microbiome that is either robust to malignant transformation or the result of systemic alterations in both tumor and adjacent normal (hepatic) tissue. This pattern is also reflected in similar tissue microbiome diversity between paired tumor and normal tissues (Fig. 2b). However, an increase in microbial diversity in OVa vs non-OVa subjects was observed (*p*-value = 0.026 for normal tissue; Fig. 2b), indicating that *O. viverrini* infection associates with an altered microbiome in the bile duct.

Comparison of microbiome profiles for normal hepatic tissues from non-CCA patients (n = 5) with adjacent normal (hepatic) tissues in CCA patients (n = 60) showed statistically significant differences (Adonis test *p*-value = 0.001 and 0.004 for unweighted and weighted UniFrac distances, respectively (OVa; n = 28); Adonis test *p*-value = 0.003 and 0.023 for unweighted and weighted UniFrac distances, respectively (non-OVA; n = 32)), in support of the existence of systemic microbiome alterations in CCA patients. At the bacterial family level, significant differences were observed for *Enterobacteriaceae* (FDR adjusted *p*-value = 0.048 (non-OVa)) and *Lachnospiraceae* (FDR adjusted *p*-value = 0.048 (non-OVa)) with borderline significance for two other families (*Sphingomonadaceae*, *Bifidobacteriaceae*; Supplementary Fig. 5). *Enterobacteriaceae* is a family of gram-negative bacteria that includes many pathogens found in the digestive tract (e.g. *Klebsiella*, *Salmonella* and *Escherichia coli*), while *Lachnospiraceae* is a family of anaerobic bacteria that are frequently found in the human gut.

Despite the systemic impact on the community, specific bacteria may colonize differently in tumor vs adjacent normal tissues in CCA patients. A comparison across all profiled bacterial genera revealed a single genus (*Stenotrophomonas*, *Xanthomonadaceae* family), to be significantly enriched in tumor vs adjacent normal for non-OVa patients (FDR adjusted *p*-value = 0.039; Supplementary Fig. 6). Interestingly, *Stenotrophomonas* has been previously implicated in bile duct infections (Perez et al., 2014). Notably, *Stenotrophomonas* was not found to significantly differ in abundance in tumor vs adjacent normal tissues for the OVa group (Supplementary Fig. 6), reflecting the distinct etiologies of the two groups.

For the OVa group, there was no significant enrichment or depletion in bacterial taxa between tumor and adjacent normal tissues (n = 28; Adonis test *p*-value = 0.599 and 0.711 for unweighted and weighted UniFrac distances, respectively). However, comparing OVa vs non-OVa tissue showed significant differences (Adonis test *p*-value = 0.001 for unweighted UniFrac distance) and enrichment for *Bifidobacteriaceae* (FDR adjusted *p*-value = 5.91 × 10^− 6^ for adjacent normal tissues and 7.80 × 10^− 7^ for tumors), *Enterobacteriaceae* (FDR adjusted *p*-value = 0.028 for adjacent normal tissues and 0.0058 for tumors) and *Enterococcaceae* (with borderline significance; FDR adjusted *p*-value = 0.060 for adjacent normal tissues and 0.056 for tumors with Fisher's combined *p*-value = 0.02; Fig. 2c). The enrichment was exceptionally strong for *Bifidobacteriaceae* (a family of anaerobic bacteria that is frequently found in the gut, vagina and oral microbiota), which was detected in 41 (out of 56 adjacent normal and tumor) OVa samples vs 5 (out of 64 adjacent normal and tumor) non-OVa samples. Since these bacteria are enriched in both adjacent normal and tumor tissues, their role in promoting tumorigenesis, if any, is likely to be mediated through a distal acting mechanism unlike that for *Fusobacterium* which is closely associated with colorectal tumor tissue (Kostic et al., 2012).

### 3.3 *Bifidobacteriaceae* Is the Dominant Member of the *O. viverrini* Microbiome

**Fig. 3:**
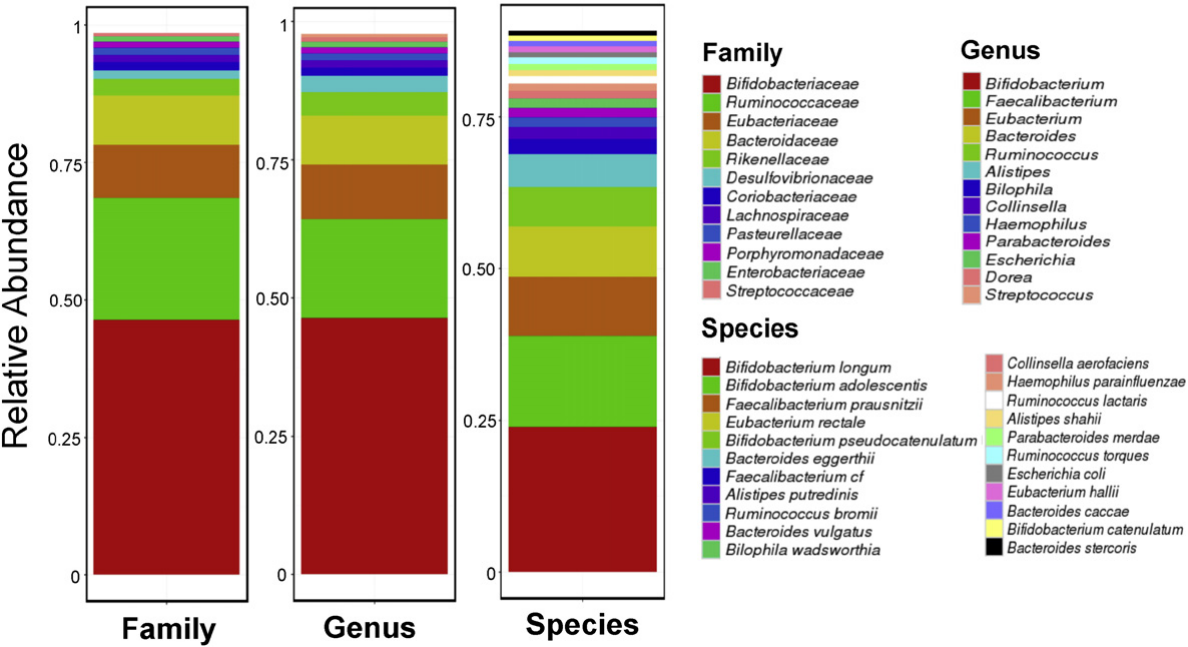
Composition of the *O. viverrini* microbiome based on whole metagenome profiling. Breakdown of the composition of the *O. viverrini* microbiome at family, genus and species levels. Only members with  > 0.5% abundance are shown.

Based on the increased microbial diversity in *O. viverrini* associated tissues (Fig. 2b), a working hypothesis is that the introduction of novel microbes into bile duct tissues could be a consequence of *O. viverrini* parasitism. To explore this, reads originating from bacterial species in a previous *O. viverrini* shotgun sequencing study (pool of flukes from an animal model of opisthorchiasis) were examined to reconstruct the *O. viverrini* microbiome (Young et al., 2014). Notably, *Bifidobacteriaceae*, the bacterial family that is highly enriched in *O. viverrini* associated bile duct tissues (Fig. 2c), was found as the main component of the *O. viverrini* microbiome (Fig. 3). However, for the other two bacterial families (*Enterobacteriaceae* and *Enterococcaceae*) which were also enriched in OVa group tissues, we noted that they were either at very low abundances (*Enterobacteriaceae*) or not detectable at our threshold (*Enterococcaceae*) in the *O. viverrini* microbiome (Fig. 3). These results suggest that additional factors could have contributed to their enrichment in OVa group tissues.

### 3.4 Metabolic Pathways Enriched in *O. viverrini* Associated Tissue Microbiomes Are Linked to Tumorigenesis

**Fig. 4:**
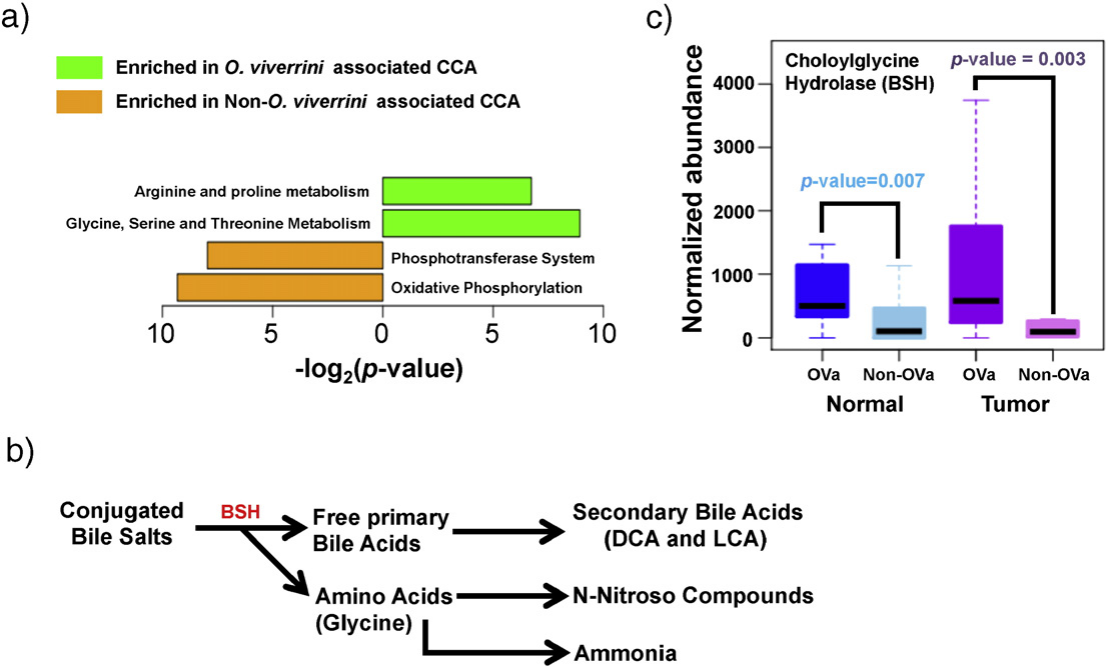
Functional analysis of the *O. viverrini* associated tissue microbiome. (a) Functional pathways that were identified to be differentially abundant in the *O. viverrini* associated (n = 28 pairs) and non-associated (n = 32 pairs) CCA tissue microbiomes. (b) Breakdown of bile salts into metabolic products implicated in carcinogenesis. (c) Boxplots of predicted abundances of the *bsh* gene in respective tissue microbiomes (OVa, n = 28; non-OVa, n = 32). All *p*-values were calculated using the Wilcoxon rank-sum test.

The metabolic output of the microbiome can directly impact tumorigenesis (Louis et al., 2014, Swartz et al., 2012). To evaluate if this is the case in CCA, pathway abundances for tissue microbiomes were reconstructed and assessed based on their 16S rRNA profiles (see Materials and Methods). These were then compared between OVa and non-OVa tissue microbiomes to determine their functional differences. Strikingly, amino acid metabolism pathways (Arginine and Proline; Glycine, Serine and Threonine) emerged as the two enriched pathways in OVa vs non-OVa tissue microbiomes (mirroring recent studies showing glycine dependency in tumor-initiating cells (Zhang et al., 2012)), while genes in the phosphotransferase system and oxidative phosphorylation pathways were enriched in non-OVa tissue microbiomes vs their OVa counterparts (Fig. 4a). Pathways enriched in non-OVa tissue microbiomes are key for energy production and understanding their functional implications for carcinogenesis would require further investigation.

*Bifidobacteriaceae*, *Enterobacteriaceae* and *Enterococcaceae*, shown earlier to be enriched in OVa tissue microbiomes, are known constituents of the gut microbiome (Fei and Zhao, 2013, Kostic et al., 2015, Matsuki et al., 1999). Metabolic activities of specific gut microbiota are known to result in the formation of carcinogens such as ammonia and bile acids (Fig. 4b), which have been implicated in colorectal cancer progression (Louis et al., 2014). The analysis of enriched microbial pathways points to a similar role for the tissue microbiome in *O. viverrini* associated CCA development. Specifically, the enrichment of amino acid metabolism pathways in the OVa tissue microbiome also increases its potential to generate ammonia as a side product (Fig. 4a). This was accompanied by significantly higher predicted (Langille et al., 2013) abundance of bile salt hydrolases (BSH) (*p*-value = 0.007 in normal tissue and *p*-value = 0.003 in tumor tissues) in OVa vs non-OVa tissue microbiomes (Fig. 4c). BSH is an important enzyme that is produced by gut bacteria to break down bile salts into primary bile acids which are further metabolized to secondary bile acids (Fig. 4b). Bile acids have been previously shown to lead to DNA damage in host cells, culminating in carcinogenesis (Yoshimoto et al., 2013). Together, these results provide a link between the altered microbiome in OVa tissues and its contribution to tumorigenesis.

## 4 Discussion

Until recently, many internal organs were believed to be sterile environments. Recent studies have however changed that view, showing that even healthy placentas consistently harbor microbial communities (Aagaard et al., 2014). This study serves to shed light on the complex microbial communities’ resident in parts of the hepatobiliary system. Crosstalk between these communities and those in the intestine could be mediated by the process of enterohepatic circulation, influencing key processes in the host such as nutrient acquisition and drug metabolism (Yip et al., unpublished).

Tumor microenvironment, defined as the assortment of host and microbial cells associated with tumors, is known to be critical in regulating carcinogenesis (Swartz et al., 2012). There has been increasing evidence that supports a role for microbiota in shaping the microenvironment through its metabolic output and interaction with host cells (Louis et al., 2014, Swartz et al., 2012). The biliary system is prone to microbial infections (Carpenter, 1998) and the interactions between bacteria and bile profoundly impact human health (Begley et al., 2005). However, little is known about the biliary tissue microbiome and its contribution to bile duct tumorigenesis. Description of the biliary tissue microbiome has been typically generalized from results originating from bile fluid cultures and may not be reflective of the biliary tissue microenvironment. This study serves to shed light on the role of the bile duct tissue microbiome in CCA development based on extensive 16S rRNA profiling of *O. viverrini* associated and non-associated paired tumor-normal tissues. To the best of our knowledge, this is the first study to profile tumor and adjacent normal tissue microbiomes of the biliary tree and serves to further our understanding of the diversity and functional capacity of the resident community, complementing earlier studies that used bile fluids or unmatched samples (Aviles-Jimenez et al., 2016, Wu et al., 2013).

Overall, we found systemic differences in tumor and adjacent normal CCA tissue microbiomes compared to normal hepatic tissue, but limited divergence in paired tumor vs normal microbiomes. In fact, *O. viverrini* associated tissues did not exhibit any significant microbiome alterations between tumor-normal pairs. The systemic alterations in CCA tissue microbiomes suggest that the tumor and tissue-resident microbiomes influence each other using far-acting mechanisms. For non-*O. viverrini* associated tissue samples, the genus *Stenotrophomonas* was found to be more abundant in tumor tissue. This is similar to the distribution profile for *Fusobacterium* in colorectal carcinoma (Kostic et al., 2012). *Stenotrophomonas* is known to be involved in bile duct infections (Perez et al., 2014) and shown to elicit proinflammatory cytokine production in vitro (Roscetto et al., 2015). Furthermore, CCA development has been strongly linked to an inflammatory phenotype (Sia et al., 2013). Consequently, an inflammation mediated mechanism tying the enrichment of *Stenotrophomonas* to non-OVa CCA tumorigenesis deserves further investigation.

Compared to non-OVa tissues, there was notable enrichment of specific enteric microbes such as *Bifidobacteriaceae* and *Enterobacteriaceae* in the OVa tissue microbiome. *Bifidobacteriaceae* was also seen as the major constituent of the *O. viverrini* microbiome (with *Enterobacteriaceae* at much lower abundance), providing a link between *O. viverrini* infection and CCA tissue microbiome alteration in humans, and extending on previous findings based on rodent models (Plieskatt et al., 2013). As *O. viverrini* infection in humans requires the consumption of infected raw fish, we hypothesize that it triggers the observed microbiome alterations and not vice versa. While an earlier study detected the presence of *Helicobacter pylori* in the bile of liver fluke infected CCA patients (Boonyanugomol et al., 2012), we did not detect *H. pylori* in our tissue samples, possibly due to the transient nature of bile fluids and their differences in comparison to bile duct tissue microbiomes as noted in this study.

Enteric bacteria have been implicated in cancer progression of the gut (Louis et al., 2014, Schwabe and Jobin, 2013). Their increased abundance in bile duct tissues could promote tumorigenesis in a similar manner. Indeed, the data in this study supports an increase in the microbial production of carcinogens such as bile acids (Yoshimoto et al., 2013) and ammonia (Louis et al., 2014) in the altered microbiome of OVa tissues. On the basis of these results, we propose a model involving (i) *O. viverrini* mediated introduction of specific enteric microbes into the bile duct, (ii) subsequent alterations in the metabolic output of bile duct tissue microbiomes, and (iii) increased levels of potentially carcinogenic metabolites, culminating in a tissue microenvironment primed towards malignant transformation. Recent data from a work that found elevated levels of bile acids in CCA patients, lends further support to this model (Jusakul et al., 2012). A recent study (Sivan et al., 2015) demonstrated a role for gut-dwelling *Bifidobacteria* in promoting systemic antitumor immunity. Further studies are thus needed to evaluate the role of increased *Bifidobacteria* in bile duct tissues with respect to OVa carcinogenesis.

Apart from *O. viverrini*, *Opisthorchis felineus* and *sinensis* *Clonorchis* are also known to infect the biliary tract. Indeed, *C. sinensis* infection is known as a risk factor for CCA (Tyson and El-Serag, 2011). Comparing microbiome alterations in biliary tissues associated with *O. viverrini* and *C. sinensis* infections could further clarify the commonalities and role of the biliary tissue microbiome in parasite-associated CCA.

Our study demonstrates that microbiomes of internal human organs are highly variable in composition but specialized to their respective distinct niches. The biological reciprocity between the human host and the tissue microbiome can influence the regulation of specific physiological processes. Taken together, the data in this study supports the notion that compositional shifts in the tissue microbiome following parasite infection can enable an altered microenvironment to drive tumorigenesis. Indeed, Bongers et al. (Bongers et al., 2014) and others have shown tumor development to be sustained by the crosstalk between the genetics of the host and the corresponding specific microbiome. The results in this work provide observational evidence for this model but further functional work is needed in the context of CCA carcinogenesis. In addition, as the study pools data from three different countries, other confounding clinical or epidemiological factors (such as the origin of OVa CCA samples in Thailand) could explain observed difference in OVa vs non-OVa CCA microbiomes. Additional samples from other regions of the world and an integrated approach that combines information about host genetics (e.g. by sequencing the tumor genome), functional output (e.g. from proteomics and metabonomics) and environmental factors (e.g. microbiome profiling) is thus likely needed to dissect host and microbiome contributions to tumorigenesis.

## Disclosure of Potential Conflict of Interest

No author had any financial or personal relationships that could inappropriately influence or bias this work.

## Author Contributions

Study concept and design: K.R.C., S.H.C., N.N., C.P., J.N.

Acquisition of data: K.R.C., S.H.C., A.H.Q.N., C.K.O., C.L., A.J., D.B., A.W., S.P.C., Z.F., K.G.Y., P.T., N.N., C.P., J.N.

Analysis and interpretation of data: K.R.C., S.H.C., C.L., N.N., C.P., J.N.

Drafting of the manuscript: K.R.C., S.H.C., N.N., C.P., J.N.

Critical revision of the manuscript for important intellectual content: All authors.

Statistical analysis: K.R.C., C.L.

Obtained funding: N.N., T.B.T., P.T., D.G.D, J.N.

Technical/material support: D.M.Y.T., K.H.L., R.S., D.G.D., S.D., I.P., C.W., P.Y., S.W., V.B., N.K., C.P.

## Appendix A Supplementary data

Supplementary Figures Image 1

Supplementary File 1 Image 2
