# Supplementary material for: Tissue Microbiome Profiling Identifies an Enrichment of Specific Enteric Bacteria in Opisthorchis viverrini Associated Cholangiocarcinoma
Source: eBioMedicine. 2016 May 6;8:195–202. doi: 10.1016/j.ebiom.2016.04.034 (PMC4919562; doi:10.1016/j.ebiom.2016.04.034)
Supplement: Supplementary file 1 — Supplementary Figures [file mmc1.pdf]

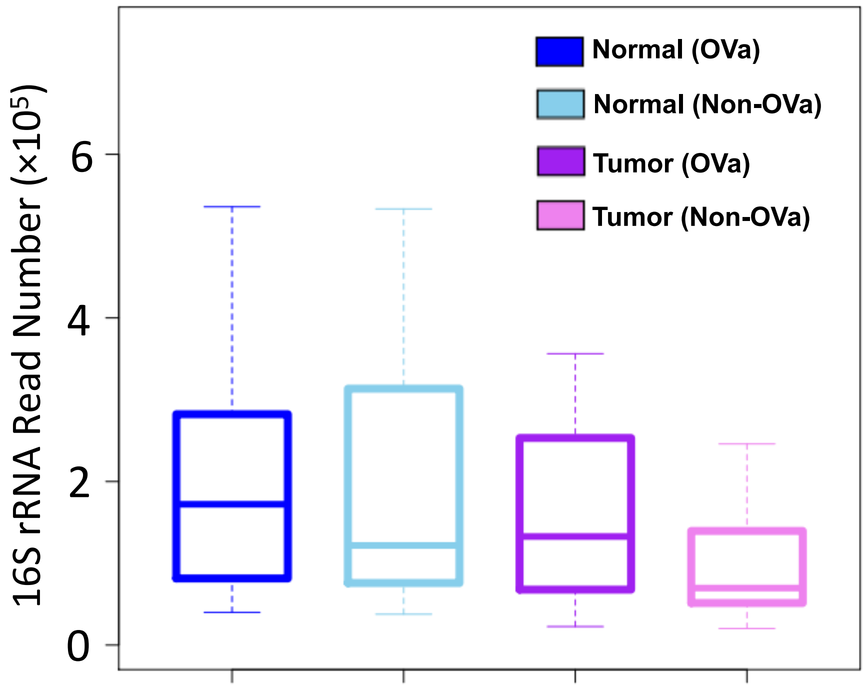

Supplementary Figure 1. Boxplots showing the number of 16S rRNA reads for different groups of samples.

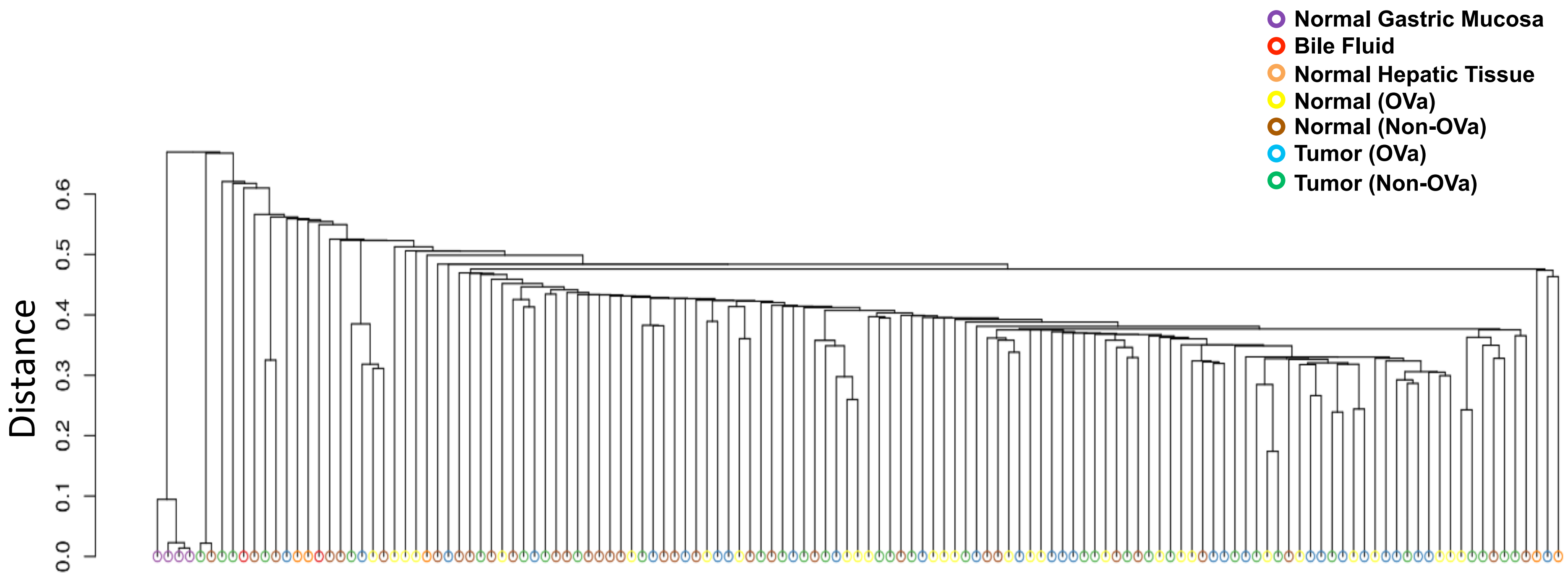

Supplementary Figure 2. Clustering of microbiome profiles from different samples. Dendrogram representation was derived from single-linkage clustering on unweighted UniFrac distances of microbiome profiles from non-cancer hepatic tissue (n=5), bile fluid from CCA patients (n=2), non-cancer gastric mucosa (n=4) and paired tumor-normal OVa (n=28 pairs) and non-OVa (n=32 pairs) samples from CCA patients. The clustering recapitulates patterns seen in the PCoA plot in **Figure 1E**, with samples from non-cancer gastric mucosa clustering together and being clearly distinct from tumor and adjacent normal tissues from CCA patients.

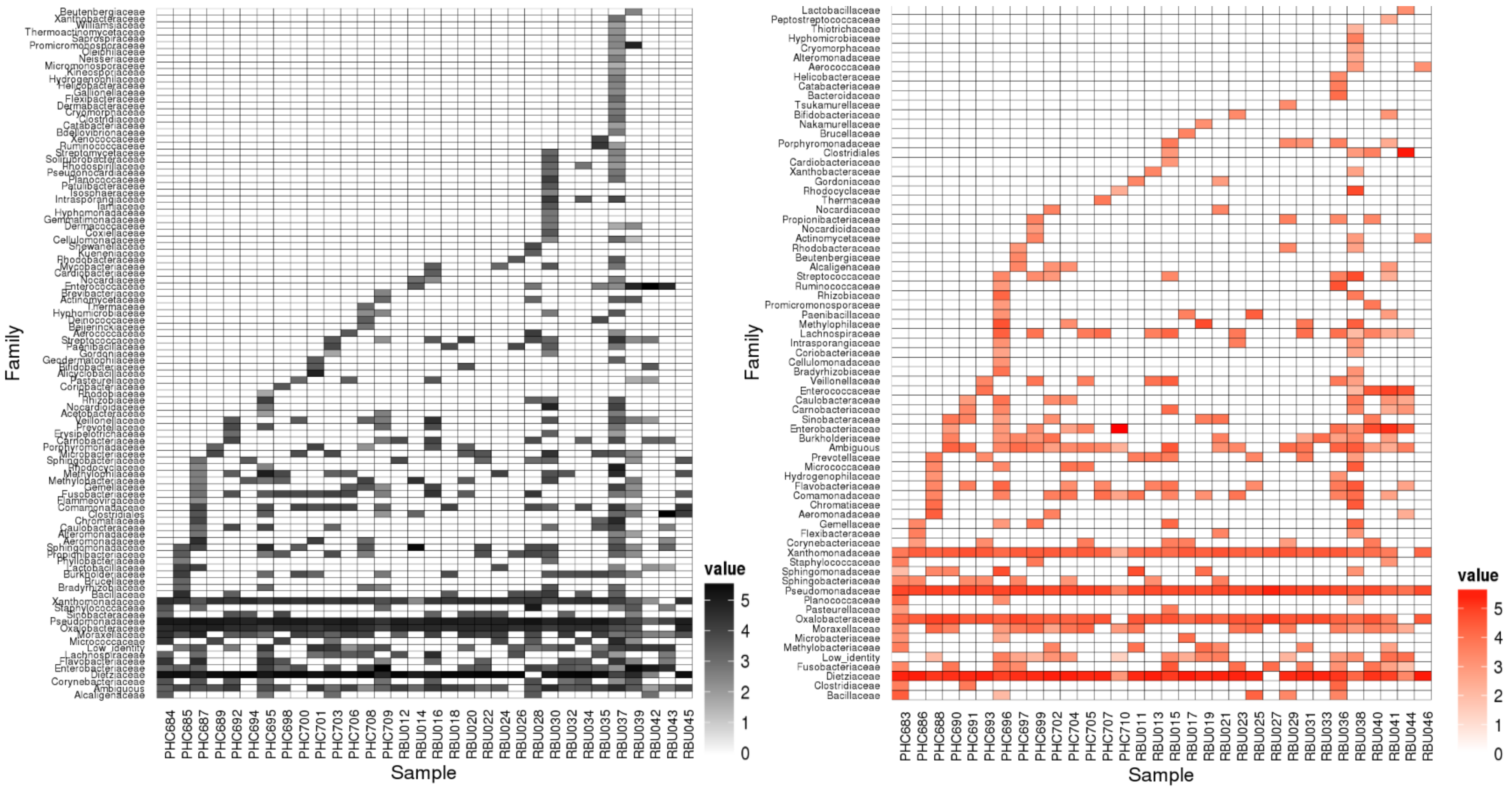

Supplementary Figure 3. Heatmaps showing logarithm of normalised 16S rRNA reads from different families in non-*O. viverrini* associated (Left) normal and (Right) tumor tissue samples (n=32).

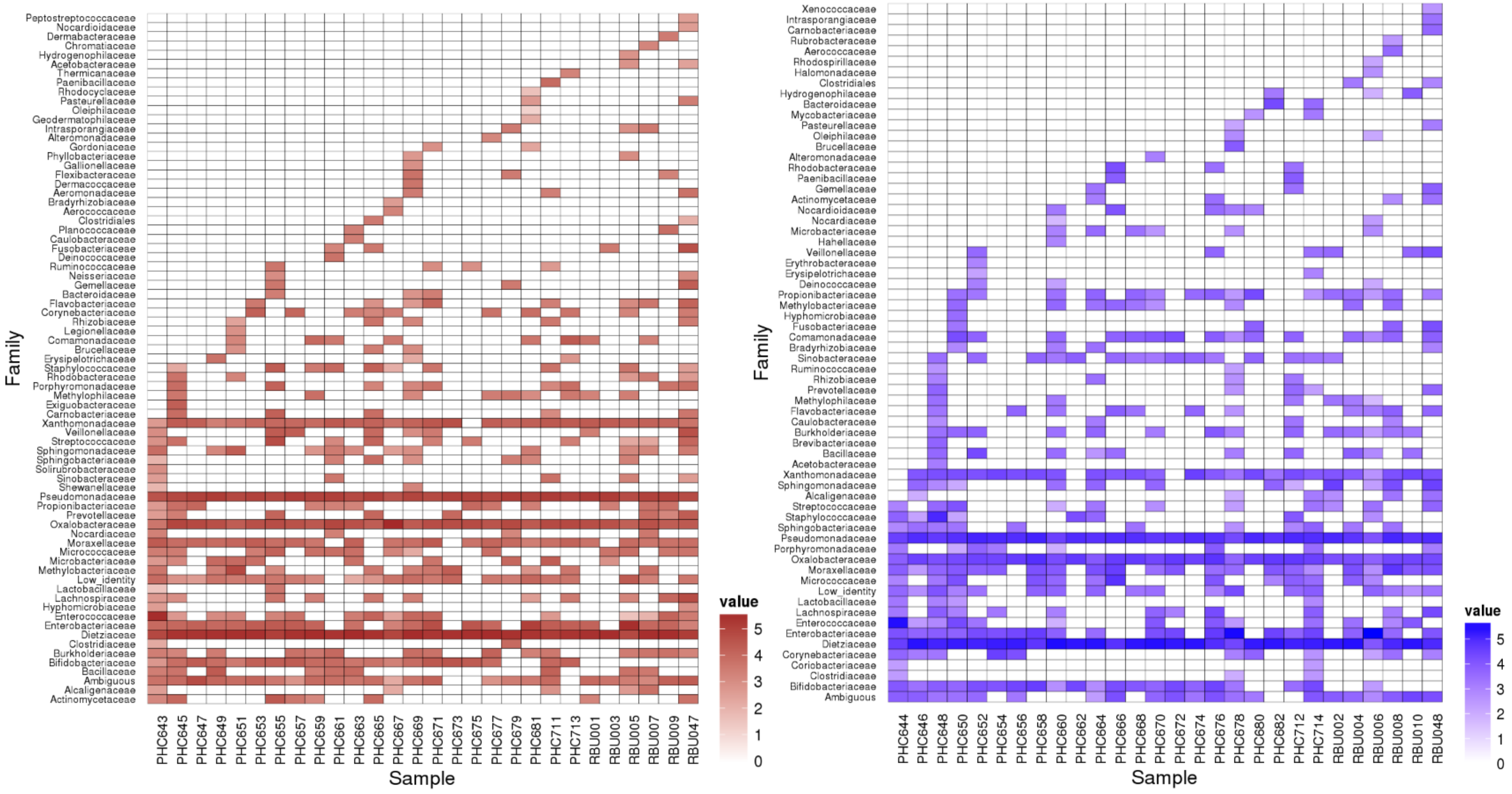

Supplementary Figure 4. Heatmaps showing logarithm of normalised 16S rRNA reads from different families in *O. viverrini* associated (Left) normal and (Right) tumor tissue samples (n=28).

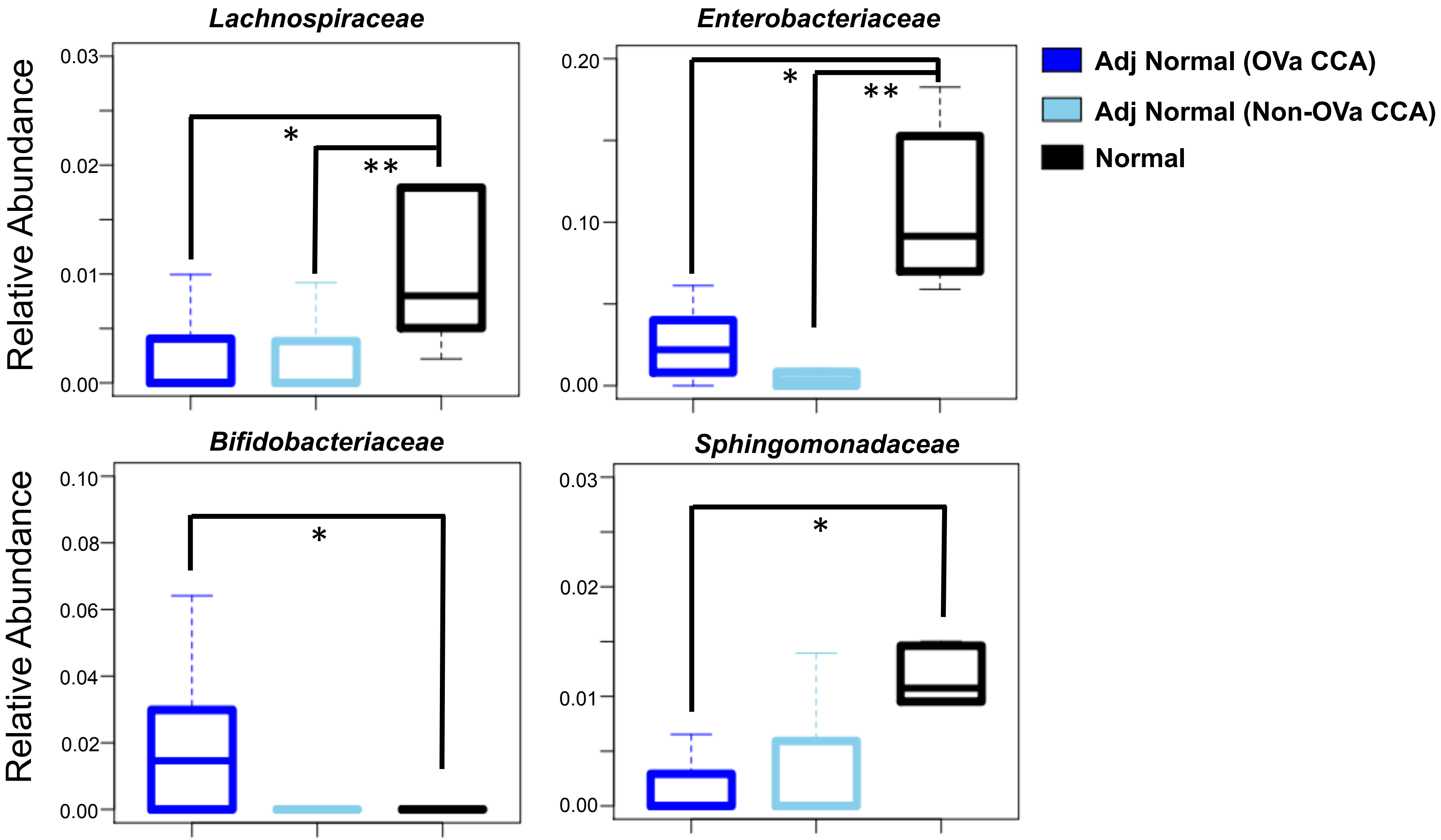

Supplementary Figure 5. Boxplots showing relative abundance of bacteria families differentially abundant in adjacent normal hepatic tissues of CCA individuals (28 OVa and 32 non-OVa) versus normal hepatic tissues (5) of non-CCA individuals. All *p*-values were calculated using Wilcoxon rank-sum test, where ** and * represent FDR adjusted *p*-value < 0.05 and 0.1 respectively.

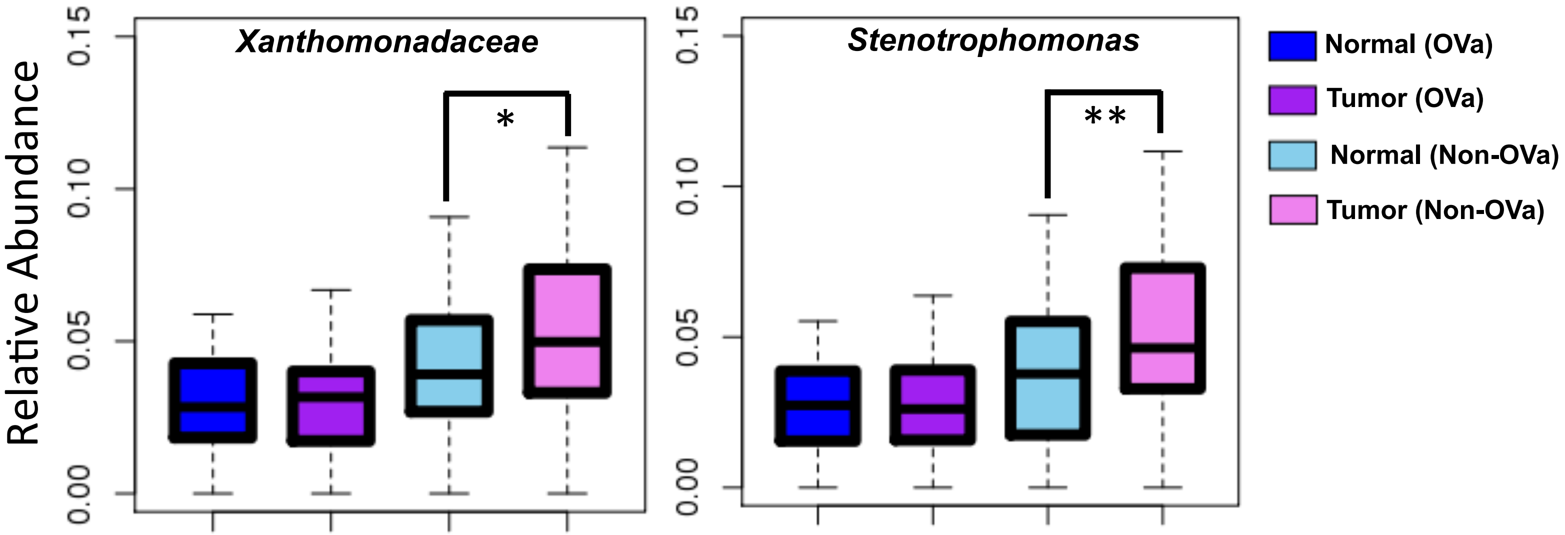

Supplementary Figure 6. Boxplots showing relative abundance of bacteria enriched in non-OVa tumor tissues versus paired normal (28 OVa and 32 non-OVa). The left plot is at the family level while the right plot is at the genus level. All *p*-values were calculated using Wilcoxon signed-rank test, where ** and * represent FDR adjusted *p*-value < 0.05 and 0.1 respectively.
